# Relationship between Anthropometric, Physical and Hormonal Parameters among Pre-Pubertal Handball Players

**DOI:** 10.3390/ijerph18199977

**Published:** 2021-09-23

**Authors:** Alexandra Cselkó, Edina Ivett Szabó, Mark Váczi, Tamas Kőszegi, Eva Tékus, Marta Wilhelm

**Affiliations:** 1 Institute of Sport Sciences and Physical Education, University of Pécs, H-7624 Pécs, Hungary; szaboedina90@gmail.com (E.I.S.); vaczi@gamma.ttk.pte.hu (M.V.); eva.tekus@aok.pte.hu (E.T.); mwilhelm@gamma.ttk.pte.hu (M.W.); 2 Doctoral School of Health Sciences, University of Pécs, H-7621 Pécs, Hungary; 3 Department of Anatomy, Medical School, University of Pécs, H-7624 Pécs, Hungary; 4 Department of Laboratory Medicine, Medical School, University of Pécs, H-7624 Pécs, Hungary; koszegi.tamas@pte.hu; 5 János Szentágothai Research Center, University of Pécs, H-7624 Pécs, Hungary; 6 Sports Medicine Center, Medical School, University of Pécs, H-7632 Pécs, Hungary

**Keywords:** handball, children, strength, sex hormones, cortisol

## Abstract

Background: The aims of our study were to investigate the changes in anthropometric and physical parameters and fasting hormonal levels among pre-pubertal female handball players (n = 14, age: 11.53 ± 0.58 yrs, height: 153.36 ± 5.12 cm, body mass: 43.59 ± 6.14 kg) in the pre-season period following 8 weeks of handball training, and to analyze the contribution of hormones, physical performance and anthropometric parameters. Methods: Prior to and immediately following the training period, several anthropometric, strength, and cardiorespiratory variables, including fasting hormonal concentrations (plasma cortisol, estradiol, testosterone and growth hormones) were measured. Athletes performed concurrent resistance and aerobic exercises, including game-based trainings during the 8-week training period. Results: Significant elevations were found in all strength parameters (maximal handgrip strength dominant (D): 16.40%, *p* < 0.01; non-dominant (ND): 25.15%, *p* < 0.05; maximal concentric (MVC) torque of quadriceps D: 13.82%, *p* < 0.05; ND: 12.61%, *p* < 0.05; MVC torque of hamstring D: 12.14%, *p* < 0.01; ND: 12.44%, *p* < 0.01), including plasma cortisol levels (C, 34.30%, *p* < 0.05) and peak respiratory quotient (5.24%, *p* < 0.05). Body composition and maximal oxygen uptake (VO_2max_) remained unchanged. Percentage changes in thigh (r = 0.316, *p* < 0.05), hand (r = 0.361, *p* < 0.05), and hip circumference (r = 0.297, *p* < 0.05) correlated with C changes. Percentage changes in plasma growth hormone levels (GH) contributed to the magnitude of gains in handgrip strength (r = 0.553, *p* < 0.05). Percentage changes in maximal exercise pulmonary ventilation (MVE) correlated with elevated C (r = −0.592, *p* < 0.05). Discussion: Changes in anthropometric variables and fasting hormone levels (estradiol, testosterone and cortisol) were poor indicators of developing VO_2max_ and strength during pre-pubertal years. Physical adaptation may not be explained in consideration of the athletes’ hormonal or anthropometric characteristics. Conclusion: Gradually increased training volume followed by a summer break should be applied to youth handball, considering the anti-hypertrophic responses and the inhibitory effect of elevating C on pre-pubertal maturation.

## 1. Introduction

During pre-pubertal years, numerous genetic, anthropometric, metabolic, neurological and functional changes are regulated by hormones, influencing the development seen in physical qualities [1,2,3]. Morphophysiological changes are related to growth and maturation, mediated by hormones [4] which affect physical performance (primarily cardiorespiratory fitness) [5]. The data of hormone responses during exercise are limited in pre-pubertal ages [1]. In this period there is large difference in biological age in children that may be related to maturity status quantified by hormonal levels and anthropometrics. This factor has an impact on the final result of sports competitions [6].

Data concerning changes in endurance and in anaerobic capacity during prepubescence are sporadic in contrast to puberty [1,7,8], but VO_2max_ elevates with age [9]. Significant improvement in VO_2max_ is not yet proven [10]; however, the data are controversial due to differences regarding training protocols and experimental designs [11]. Interestingly, pre-pubertal children and well-trained endurance athletes have similar metabolic responses to submaximal exercise [12,13]. In contrast to VO_2max_, the increases in muscular strength and endurance are more prominent during the childhood years [14]. In youth, strength improvement and muscle hypertrophy are based on the complex mechanisms of neural and hormonal changes [2], in which other biomechanical factors, such as levels of neuromuscular activation, changes in intrinsic contractile characteristics of muscle and improved motor coordination [15] may contribute. Hormone-determined muscle hypertrophy may not be the main factor associated in strength development during pre-pubertal years. Strength gain is largely neural with learning components, yet independent of changes in muscularity [16]. However, little information is available concerning sex hormone level changes and their contribution to strength trainability during puberty [17]. It is well known that different hormone levels (estradiol, testosterone, growth hormone, cortisol) regulate maturation and stress responses in pre-pubertal athletes [18], but the association between the magnitude of hormonal changes and physical response must be clarified for individualizing and optimizing training programs in this age-group.

The popularity of team handball, which is an Olympic sport, has increased during recent decades in both genders [19], even if there are less female athlete-related studies than male [20]. Modern handball is a complex and multi-factoral sport game, hence the determination of the performance factors is difficult [21]. In this game, players are exposed to high-load training stimuli provided by resistance, speed, endurance, and coordination trainings [22]. There are a limited number of studies on the enrolling of pre-pubertal females and investigations into the potential factors which indicate the physical performance of youth handball athletes. Measured parameters are often the actual anthropometric and physical characteristics [23] or sex differences [22]. In the majority of research, males [24,25] who were older than preadolescent children [26,27] were examined. Furthermore, the focus of interest was mainly on the effects of handball training on motor skills [28]. Information regarding the physical performance of females during pre-pubertal ages and the influencing factors of improvement are sporadic, especially among handball players.

The aims of our study were (i) to investigate the changes in anthropometric and physical parameters (strength, VO_2max_) and fasting hormonal levels (plasma cortisol, testosterone, estradiol and growth hormones) among pre-pubertal female handball players following an 8-week handball training period, and (ii) to analyze the changes in hormones upon anthropometric parameters influencing the physical performance of athletes.

It is hypothesized that there are relationships between the changes in hormonal, anthropometric and physical parameters (mainly force development) after the training period of pre-pubertal handball players.

## 2. Materials and Methods

Procedure, participants and training:

Fourteen pre-pubertal female handball players (between 10 and 12 years old) were examined in our study. One week before the assessments, each subject was familiarized with the testing procedures (vita maxima spiroergometric test and strength measurements). Categorization of participants was based on chronological age and breast development, referencing Tanner’s classification [29]. Tanner’s methods were used among female athletes [30,31]. Participants were classified on a five-scale Tanner stage for breast development by a nurse resident in the laboratory experienced in this technique [32]. The menarche status was self-reported. Females were in Tanner stage I (B1, based on breast development) and did not experience menarche.

All players underwent training at the same Hungarian Handball Club for at least 4 years, and participated in National Championships hosted by the Hungarian Handball Federation. In general, the players participated in five handball training sessions per week (average training time 7.5 h/week).

**Figure 1:**
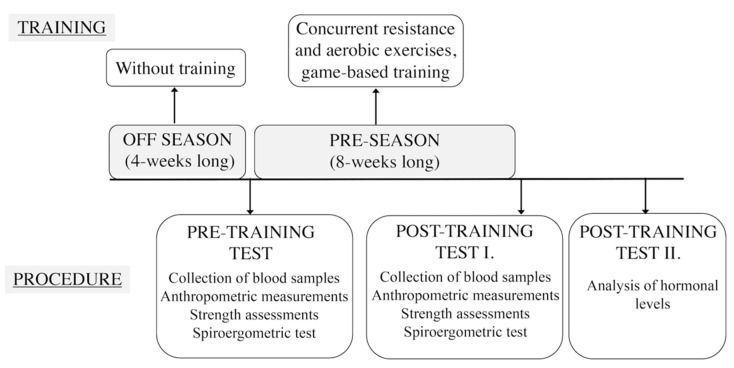
Exercise bout and measurements performed during the experiment.

The first test session (pre-training) was performed when players began their pre-season period and training following a 1-month break during the off season. The second test session (post-training) was implemented 8 weeks later. Players performed concurrent resistance and aerobic exercises, including game-based trainings (5 training sessions/week, 90 min practice/sessions) during the 8-week training period (Figure 1). During this season, the aims were to improve aerobic (duration: 25–30 min, >80% intensity of maximal heart rate) [33] and anaerobic endurance and muscle strength. Mostly training consisted of continuous running equipped with a heart rate monitor, medicine ball drills, and functional strength drills were included during the conditioning phase. The game-based training phase included fundamental drills, such as dribbling, passing, shooting, offensive and defensive footwork, individual and team tactics.

The collection of blood samples was conducted in all test sessions (later analyzing hormonal levels, refer to Section 2.4). Anthropometric measurements, strength assessments and a Bruce treadmill protocol with spiroergometric tests were conducted to collect metabolic gas-exchange parameters (Figure 1). Data collections were performed at same time of the day, under the same conditions for every subject.

All protocols and procedures were approved by the University Ethics Committee (Permit number: 5023) and were performed in full accordance with the ethical standards of the Helsinki Declaration. Subjects and their parents freely gave their consent to participate in the study.

### 2.1. Anthropometric Measurements

All anthropometric measurements were performed on the right side of the body and by the same investigator, consistently using the same devices. Body circumferences (forearm, hand, hip and thigh) were assessed with an anthropometric tape (Seca 201, Seca Corporation, Hamburg, Germany, accuracy of 0.1 cm), using the standardized techniques of The International Society for the Advancement of Kinanthropometry [34]. Anthropometric variables, such as circumferences, highly correlated with muscle mass in children, suggesting a relationship between the muscle volume and circumferences [35].

Body height (Martin type anthropometer), body mass (Beurer BG-55 scale, Beurer GmbH, Ulm, Germany) of the participants were measured and body mass index (BMI) was calculated. Body fat percentage was estimated from the triceps and the calf skinfold thicknesses (Lange caliper, Model SH5020, Saehan Corporation, Masan, South Korea), using equations associated with Slaughter et al. (1988) [36], for girls.

### 2.2. Strength Measurement

All players performed a five-minute aerobic warm-up on a cycling ergometer (Ergoline 900, Ergometrics, Bitz, Germany) and five minutes stretching of the knee extensors and hip flexors prior to the assessment. Next, bilateral maximal isometric concentric quadriceps and hamstring torque (on both sides) were measured at 70° knee angle using a dynamometer (Multicont II device, Mediagnost and Mechatronic Ltd., Budapest and Szeged, Hungary) while in a seated position.

Handgrip strength was also assessed using a hydraulic hand dynamometer (Model SH5001, Saehan Corporation, Masan, South Korea) for both the dominant and the non-dominant hands [37]. In both strength tests, players performed three trials with 1 min rests between trials and the highest values were considered.

### 2.3. Spiroergometric Measurement

To assess the cardiorespiratory variables among players, a vita maxima spiroergometric test was used with the Bruce treadmill protocol [38]. The treadmill (TMX 42, Full Vision Inc., Newton, KS, USA) began at 2.74 km/h with a gradient of 10%. At 3-min intervals, both the gradient and speed were simultaneously increased. The heart rate (HR) was monitored using a 12-lead electrocardiograph (CardioVIT AT-104, Schiller AG, Baar, Switzerland) and metabolic gas-exchange parameters were continuously measured using a gas analyzer (PowerCube-Ergo, Ganshorn Medizin Electronic GmbH, Niederlauer, Germany), initiating a breath-by-breath method. Peak respiratory exchange ratio (peak RER), maximal exercise pulmonary ventilation (MVE) and VO_2max_ during the exercise test were also recorded. Two criteria were needed to finish the vita-maxima test for the completion of the following three, valid VO_2max_ which were determined: HR ≥ 195 bpm, RER > 1.0 and volitional fatigue [39].

### 2.4. Hormonal Levels

Fasting and resting morning (a.m. 7.00–8.00) venous blood samples were collected 5 days after the last training session in EDTA containing tubes (in each case, 2 × 3 mL) from antecubital vein prior to and followed by the 8-week training program. Following centrifugation (1500× *g*, 10 min), the supernatant plasma was stored in Eppendorf tubes (3 × 2.0 mL) at −70 °C until further application. Hormone levels were measured by a routine automated chemilumnescent immunoassay using standard laboratory procedures (Immulite 2000, Siemens Healthcare GmbH, Erlangen, Germany). Chemiluminescent detection was used to measure the concentration of plasma estradiol, cortisol, testosterone and growth hormone levels.

### 2.5. Statistical Analyses

The normality of data was evaluated using a Kolmogorov–Smirnov test. Each variable depicted normal distribution; therefore, differences among the variables of the two tests (pre-training and post-training) were analyzed by a paired t-test. For every variable, pre-to-post percent changes were calculated. To investigate the relationship between the percentage changes regarding the variables, the Pearson correlation test was used. The value of the correlation coefficient between 0.00 and 0.10 implied a negligible relationship and the value from 0.10 to 0.39 described a weak correlation. The range of moderate correlation was between 0.40 and 0.69, while strong correlation was between 0.70 and 0.89. Correlation coefficient’s range from 0.90 to 1.00 shows a very strong correlation. Positive correlation could be considered as the changes between the two variables in the same direction, while negative correlation was a difference in the opposite direction [40]. The test–retest reliabilities of variables were analyzed using intra-class correlation coefficients, while the test–retest differences were determined using matched-pairs t-tests. ICCs for anthropometric variables ranged from 0.90 to 0.98, ICCs of strength parameters were in the range of 0.85–0.96 and ICCs of hormonal levels ranged between 0.81–0.95. Differences in all variables of the test–retest were negligible. All mentioned assessments showed high reliability. Cohen’s d values were calculated by the measured variables to quantify effect sizes (Table 1 and Table 2) and d values were interpreted based on Cohen (1988) [41].

Values are reported as the mean, standard deviation (STD) and standard error of mean (SEM). The level of statistical significance was set at *p* < 0.05.

## 3. Results

### 3.1. Percentage Changes in Anthropometric, Physical and Hormonal Variables

Table 1 shows the percentage changes in physical attributes, anthropometric and cardiorespiratory parameters among handball players. The variables of age (*p* = 0.0001); height (*p* = 0.0001); forearm (*p* = 0.033), hip (*p* = 0.009) and thigh circumference (*p* = 0.0001); maximal handgrip strength of both sides (dominant: *p* = 0.0001, non-dominant: *p* = 0.001); maximal concentric quadriceps (dominant: *p* = 0.018, non-dominant: *p* = 0.049); hamstring torque (dominant: *p* = 0.002, non-dominant: *p* = 0.006), and peak RER (*p* = 0.001) significantly increased during the training period.

We did not find significant changes in body mass, body fat percent, BMI, VO_2max_, or MVE immediately following the test and maximal HR. There was no change among most measured hormonal variables (estradiol, testosterone, growth hormone levels and testosterone/cortisol ratio), except C, which was significantly elevated during the 8-week training period (*p* = 0.033, Table 2).

### 3.2. Correlations between Physical Characteristics, Anthropometric and Cardiorespiratory Parameters and Hormonal Levels

Despite the fact that all body segment circumferences including quadriceps and hamstring torque (on both sides) significantly increased, the percentage changes among these variables were not correlated.

**Figure 2:**
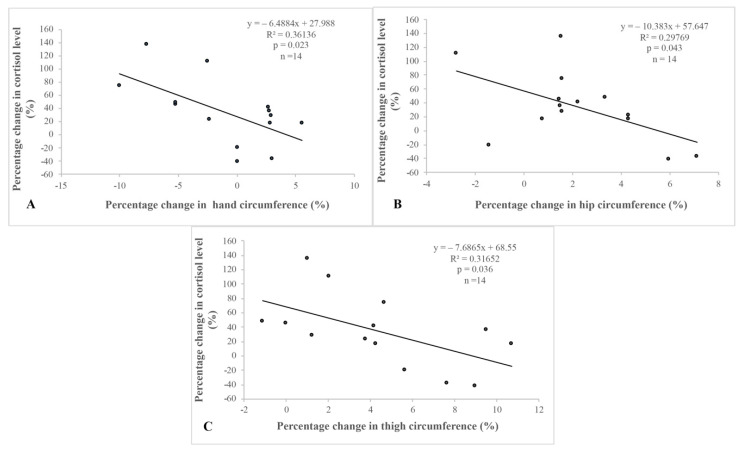
Correlations between percentage changes in anthropometric parameters and in cortisol levels. There was a significant correlation between (**A**): the percentage change in hand circumference and plasma cortisol level, (**B**): the percentage change in hip circumference and plasma cortisol level, and (**C**): the percentage change in thigh circum-ference and plasma cortisol level

There was a negative correlation between percentage changes in thigh, hand and hip circumference and cortisol levels (Figure 2).

There was a significant correlation between A: the percentage change in hand circumference and plasma cortisol level, B: the percentage change in hip circumference and plasma cortisol level, and C: the percentage change in thigh circumference and plasma cortisol level.

The percentage change of dominant handgrip strength positively (moderately) correlated with the change of GH (Table 3, Figure S1). There was no correlation between the changes of strength data and the other hormone levels.

Table 4 depicts the relationship between changes in cardiorespiratory variables and blood hormonal levels. A negative, moderate correlation was found between the percentage change in MVE and in cortisol levels (Table 4, Figure S2). The percentage change in VO_2max_ did not correlate with any of the hormone level changes measured in this study (estradiol, testosterone, growth hormone and cortisol).

## 4. Discussion

Our research focused on the changes of anthropometric, physical parameters and hormonal levels and the relationship between the percentage changes in these variables among pre-pubertal, female handball players following an 8-week training period.

In our study, body composition and VO_2max_ were unchanged, while all strength parameters and C were significantly elevated following the training period. Negative correlations were found between percentage changes in circumferences and C, including percentage changes in GH and the magnitude of handgrip strength gains. Another aspect of the results could be the relationship with the over-reaching state among prepubertal female handball players in our study.

### 4.1. Percentage Changes in Anthropometric, Physical and Hormonal Variables

Anthropometric characteristics, primarily height, progressively changed at or during pubertal ages [42]. Interestingly, body height, including forearm, hip, and thigh circumference increased in the span of 8 weeks. Three circumferences (biceps relaxed, biceps flexed and thigh circumferences) were elevated among prepubescent males and females in the experimental group after a 12-week strength training program [43]. As expected, BMI and body fat percent were lower than the mean values of non-athletes at this age, based on the research of Cole et al. (2000) [44] and Marques-Vidal et al. (2008) [45] in both pre- and/or post-training.

The handgrip strength measurement was widely used as a handball-specific physical test [37], including in clinical prevention and treatment options [46]. Based on our results, significant improvements were found in both the maximal handgrip strength (on both sides) and maximal concentric torque (quadriceps and hamstring), following the training period. In pre- and post-training assessments, the dominant handgrip strength among handball players was higher, compared to the mean reference values of 12-year-old students from Hungary [47]. Reference data regarding maximal concentric torque (quadriceps and hamstring) were not found among young, female handball players. De Ste Croix et al. (2009) [7] did not find differences between pre-pubertal boys and girls in the peak torque. The lack of strength data among pre-pubertal females, especially handball players, is the consequence of research gaps, since most of the studies focused on other age groups representing females [2,23] or males [26,37]. The measured concentric torque values (quadriceps and hamstring) of pre-pubertal, female handball players will provide a baseline for further studies.

Peak RER was significantly higher, meanwhile, other cardiorespiratory variables (VO_2max_, MVE, maximal HR) did not change, which was similar to the results of Williams et al. (2000) [32]. Only a few studies investigated gas-exchange parameters (mainly RER) among pre-pubertal female athletes. The peak RER was monitored by Welsman et al. (1997) [48] among schoolgirls (ten and eleven years old) and they did not find a significant difference in this ratio following an 8-week aerobic training program. Differences measured in peak RER may be the consequence of the different training protocols and population.

VO_2max_ is one of the most common components of physical performance assessment, while it is also a health-related parameter in preadolescence. Baquet et al. (2003) [11] described, 5–6% VO_2max_ improvement following aerobic training (with a different time and type) among children and adolescents. A study of a pre-pubertal group [10,49] focused on the VO_2max_ development affecting concurrent training using field measures (20 m shuttle run test) and found significantly higher VO_2max_ following an 8-week training program, contrary to our results. These results were likely explained in consideration of the different methods and subjects recruited to determine VO_2max_. Pre-pubertant, female handball players were not enrolled to monitor metabolic gas-exchange parameters (e.g., VO_2max_) within fixed, laboratory conditions. Combined high-intensity aerobic training [11] and concurrent training were more effective among pre-pubertal ages to improve VO_2max_ and strength at the same time [10].

In our study, MVE and maximal HR did not improve, suggesting unchanged respiratory functions and too brief of a training period for ideally suitable adaptation.

During the analyses of cortisol, growth hormones, estradiol and testosterone levels, we registered a significant increase. Only C. Kirwan et al. (1988) [50] described how the higher level of cortisol may be the marker of an acute physiological strain, hence, in our studied population, increased C indicated it was the normal response to the stress of a strenuous training period, not an overtraining state. Notably, typical symptoms regarding overtraining syndrome [51] were not found among our participants, nor their physical performance decrements, nor was a decrease in testosterone/cortisol ratios detected during the examined period. In addition, unchanged serum testosterone levels were found in resting conditions.

There was no significant alternation during pre- and post-training regarding estradiol and testosterone levels among female handball players, indicating they did not reach pubertal, hormonal maturation during the studied period. The measured effects of training were influenced by an altered sexual steroid and growth hormone responses, modifying stress hormone concentrations and effects. Endogenous cortisol secretion of females (even in the physiological range) determined the time of pubertal events [52], in which glucocorticoids may inhibit gonadal hormone secretion through the hypothalamus pituitary–gonadal axis [53], including growth hormone production [54]. Thus, cortisol levels may attenuate the secretion of estradiol, testosterone and growth hormones among female handball players.

Eliakim and Nemet (2013) [55] hypothesized that components of the GH/IGF-I axis (such as the IGF-1 level) were sensitive to energy balance; therefore, performing a longer period of training could relate to stable or elevated GH and IGF-I concentrations. Similarly, regarding this result, unchanged GH were assessed following an 8-week training period in the present study. This phenomenon may contribute to later pubertal maturation among females, associated with other factors differing from the regular population in a variety of sports [56].

Further research is needed to delineate the interactions among hormones (hypothalamus pituitary–gonadal, growth hormone axes and glucocorticoids) related to training during growth among the youth.

### 4.2. Correlations between Physical Characteristics, Anthropometric and Cardiorespiratory Parameters and Hormonal Levels

Increased changes in handgrip strength were associated with an elevated change in GH. Similarly, much as in the case regarding puberty [17], GH may play an important role towards an increase in muscle strength during pre-pubertal years, especially in the muscles of the upper limbs. The level of strength improvement did not correlate with changes in sex hormones and cortisol concentrations, suggesting that the measured fasting hormonal parameters (except GH) may not be indicators of strength development among preadolescence athletes. Seemingly, GH, rather than estradiol and testosterone, was sensitive to the present training protocol.

Strength gains in handgrip, the quadriceps femoris muscle and hamstring were independent of muscle cross-sectional area changes using anthropometric circumferences. Meanwhile, these circumferences and strengths significantly changed during the training period among handball players. Increases in circumferences (forearm, hip and thigh circumferences), in maximal strength (handgrip, concentric torque of the quadriceps femoris muscle and hamstring), and a nearly unchanged body fat percentage and body mass indicate that muscle mass elevation (muscle hypertrophy) was not parallel to strength gain.

In this study, there were no correlations between percentage changes in anthropometric parameters and physical characteristics (cardiorespiratoric and strength data). In agreement with Visnapuu and Jürimäe (2009) [57], we suggested that anthropometric variables were poor indicators of trainability among pre-pubertal, female handball players.

While strength improvement did not correlate with changes in anthropometric variables, there was a significant relationship between percentage changes in some anthropometric circumferences and fasting C. The elevated C could reduce the circumferences of the thigh, hand and hip. Presumably, cortisol may have an indirect anti-hypertrophic effect on major muscle groups of the body. Cortisol has a catabolic and inhibitory effect in protein synthesis which has long been known. Duclos et al. (2007) [58] summarized the negative effects of prolonged hypercortisolism as damaged microbial killing capacity, muscle catabolism, bone demineralization, anti-reproductive effects, depression and anxiety. Due to the lower number of participants, further research should be applied to investigate the reasons of the correlation between the circumferences and the cortisol level. Moreover, C was related to pulmonary functional (as forced expiratory volume in 1 s) decline among adult males [59]. In this study, there was a negative association between percentage changes in MVE and in C. Interestingly, this relationship was not described earlier among pre-pubertal, female athletes.

Finally, the main strength of our research was the investigation the influencing effects of anthropometric parameters or hormonal changes in the physical performances of female, pre-pubertal handball players, since this age group and gender had previously received little to no attention in this respect. Overall, changes in anthropometric variables and fasting hormone levels (estradiol, testosterone and cortisol) were poor indicators of developing VO_2max_ and strength during pre-pubertal years. Physical adaptation may not be explained in consideration of the athletes’ hormonal or anthropometric characteristics.

A limitation of this work was that a control group was not applied to determine the effects of the 8-week concurrent training period on measured variables; however, this was not the current purpose of this study. Consequently, we examined the cumulative effect of concurrent training and natural strength development during growth. Another limitations of this research effort weas not to measure the electrical activity of the skeletal muscle in order to examine the changes in neural function.

## 5. Conclusions

We found changes in maximal strength (handgrip and the concentric torque of quadriceps and hamstring) which were consequences of regular training in pre-pubertal ages. However, the VO_2max_ did not change with training. Elevated cortisol levels may be the normal response to stress during a strenuous training period, but not a legitimate overtraining state. Therefore, cortisol levels may attenuate the secretion of estradiol, testosterone and growth hormones, including the pubertal timing among female handball players.

We suggested that the changes in anthropometric variables and fasting hormone levels (estradiol, testosterone, growth hormones, cortisol) were poor indicators of developing VO_2max_ and strength during pre-pubertal years. We believe that a gradual increase in training volume, preferably following a summer break, should apply in handball training schedules for young people, considering the possible anti-hypertrophic responses and the inhibitory effect of elevated cortisol levels on pre-pubertal maturation.

## Figures and Tables

**Table 1:** Anthropometric parameters, physical characteristics and cardiorespiratory variables among handball players prior to and following the 8-week training period.

	Pre-Training			Post-Training			ES	Mean Changes (%)
	MEAN	STD	SEM	MEAN	STD	SEM		
Age (yrs)	11.53 **	0.58	0.15	11.70 **	0.58	0.15	infinity	1.51
Height (cm)	153.36 **	5.12	1.37	154.57 **	5.00	1.34	1.251	0.80
Body mass (kg)	43.59	6.14	1.64	43.62	5.77	1.54	0.026	0.23
Body fat percentage (%)	21.00	5.72	1.29	19.35	4.28	1.19	0.414	−5.67
BMI (kg/m^2^)	18.47	1.80	0.48	18.21	1.70	0.45	0.467	−1.34
Forearm circumference (cm)	21.00 *	2.10	0.56	21.50 *	1.51	0.40	0.641	2.73
Hip circumference (cm)	66.50 *	4.06	1.08	67.96 **	4.04	1.08	0.814	2.25
Thigh circumference (cm)	45.25 **	3.87	1.03	47.21 **	3.71	0.99	1.278	4.46
Hand circumference (cm)	18.71	1.14	0.30	18.50	0.88	0.23	0.240	−0.97
Maximal handgrip strength (kg)								
Dominant	16.64 **	2.47	0.66	19.79 **	3.17	0.85	1.339	16.40
Non-dominant	14.21 *	2.55	0.68	17.64 *	3.69	0.99	1.206	25.15
Maximal concentric torque of quadriceps (Nm)								
Dominant	71.77 *	14.62	3.91	83.31 *	14.38	3.84	1.031	13.82
Non-dominant	74.76 *	17.08	4.57	85.07 *	18.69	5.00	0.733	12.61
Maximal concentric torque of hamstring (Nm)								
Dominant	39.85 *	6.41	1.71	44.22 *	8.96	2.40	0.790	12.14
Non-dominant	38.41 *	6.47	1.73	42.90 *	11.76	3.14	0.674	12.44
Cardiorespiratory variables								
peak RER	1.03 *	0.04	0.01	1.08 *	0.04	0.01	1.127	5.24
VO2max (mL/kg/min)	44.29	4.66	1.25	44.41	3.98	1.06	0.026	0.96
MVE (L/min)	61.08	9.93	2.65	61.97	7.96	2.13	0.143	2.38
Maximal HR (bpm)	195.07	10.74	2.87	194.79	9.13	2.44	0.044	−0.06

Abbreviations: BMI: Body mass index, peak RER: Peak respiratory exchange ratio, VO_2max_: Maximal oxygen uptake, MVE: Maximal exercise pulmonary ventilation, BL: Blood lactate level, HR: Heart rate. MEAN, STD: Standard deviation, SEM: Standard error of mean, ES: Effect size. * Significant difference (*p* < 0.05) between pre- and post-training values. ** Significant difference (*p* < 0.01) between pre- and post-training values.

**Table 2:** Fasting hormonal changes in plasma estradiol, testosterone, growth hormone, and cortisol levels prior to and following the training period.

	Pre-Training			Post-Training		ES		Mean Changes (%)
	MEAN	STD	SEM	MEAN	STD	SEM		
Plasma estradiol level (pg/mL)	57.34	61.53	16.45	47.58	32.64	8.72	0.208	41.59
Plasma testosterone level (nM/L)	526.36	294.38	0.08	610.57	304.21	0.08	0.520	32.34
Plasma growth hormone level (ng/mL)	3.52	3.10	0.83	2.49	2.88	0.77	0.213	54.14
Plasma cortisol level (nM/L)	340.39 *	168.06	44.91	423.76 *	199.34	53.28	0.640	34.30
Testosterone/cortisol ratio	2.04	1.73	0.0005	1.68	1.03	0.0003	0.303	6.62

MEAN, STD: Standard deviation, SEM: Standard error of mean, ES: Effect size. * Significant difference (*p* < 0.05) between pre- and post-training values.

**Table 3:** Correlations between percentage changes in strength and hormonal levels.

	Percentage Change in Estradiol Level (%)	Percentage Change in Testosterone Level (%)	Percentage Change in Growth Hormone Level (%)	Percentage Change in Cortisol Level (%)
Percentage changes in dominant handgrip strength (%)	r = 0.124 *p* = 0.672	r = −0.362 *p* = 0.203	r = 0.553 *p* = 0.040 *	r = −0.494 *p* = 0.073
Percentage changes in non-dominant handgrip strength (%)	r = 0.298 *p* = 0.302	r = 0.173 *p* = 0.555	r = 0.114 *p* = 0.698	r = −0.065 *p* = 0.827
Percentage changes in quadriceps femoris torque (%)	r = 0.085 *p* = 0.772	r = 0.088 *p* = 0.765	r = −0.303 *p* = 0,293	r = −0.310 *p* = 0.281
Percentage changes in hamstring torque (%)	r = −0.029 *p* = 0.922	r = −0.164 *p* = 0.575	r = 0.269 *p* = 0.353	r = 0.208 *p* = 0.475

* *p* < 0.05.

**Table 4:** Correlation between percentage changes in cardiorespiratory variables and blood hormonal levels.

	Percentage Change in Estradiol Level (%)	Percentage Change in Testosterone Level (%)	Percentage Change in Growth Hormone Level (%)	Percentage Change in Cortisol Level (%)
Percentage change in peak RER (%)	r = −0.374 *p* = 0.188	r = −0.407 *p* = 0.754	r = −0.092 *p* = 0.754	r = −0.017 *p* = 0.953
Percentage change in VO_2max_ (%)	r = 0.004 *p* = 0.988	r = 0.027 *p* = 0.927	r = 0.027 *p* = 0.927	r = −0.348 *p* = 0.223
Percentage change in MVE (%)	r = −0.171 *p* = 0.559	r = −0.159 *p* = 0.587	r = −0.159 *p* = 0.587	r = −0.592 *p* = 0.026 *
Percentage change in maximal HR (%)	r = 0.138 *p* = 0.639	r = −0.282 *p* = 0.328	r = −0.282 *p* = 0.328	r = −0.486 *p* = 0.078

Abbreviations: peak RER: Peak respiratory exchange ratio, VO_2max_: Maximal oxygen uptake, MVE: Maximal exercise pulmonary ventilation, BL: Blood lactate level, HR: Heart rate. * *p* < 0.05.

## Data Availability

The data presented in this study are openly available in FigShare at https://doi.org/10.6084/m9.figshare.14792034.v1.

## Supplementary Materials

The following are available online at https://www.mdpi.com/article/10.3390/ijerph18199977/s1.
