# Supplementary figures and images for: Relationship between Anthropometric, Physical and Hormonal Parameters among Pre-Pubertal Handball Players
Source: Int J Environ Res Public Health. 2021 Sep 23;18(19):9977. doi: 10.3390/ijerph18199977 (PMC8507650; doi:10.3390/ijerph18199977)

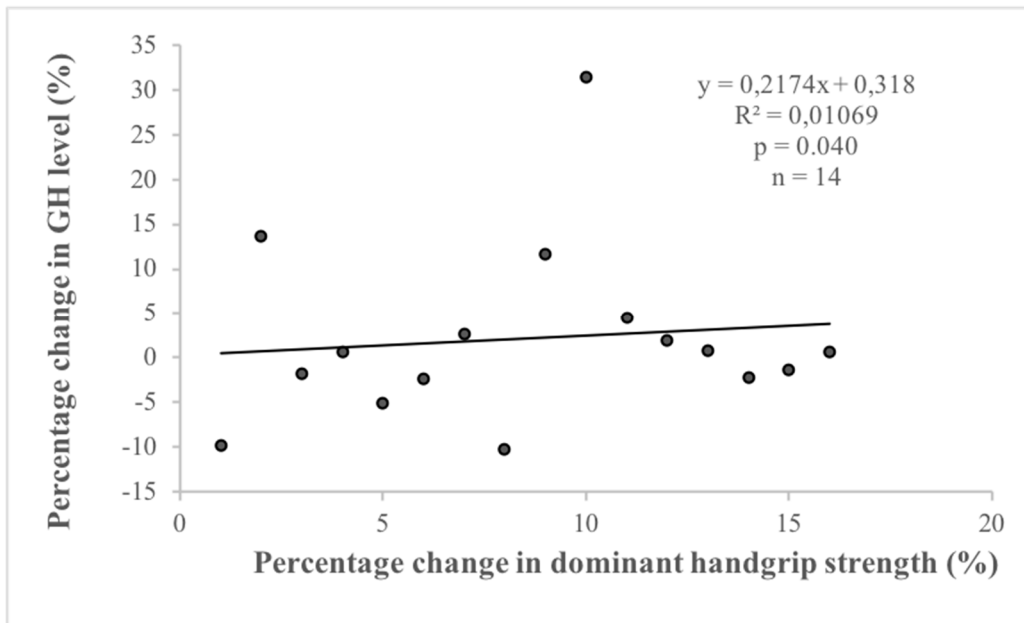

Figure S1

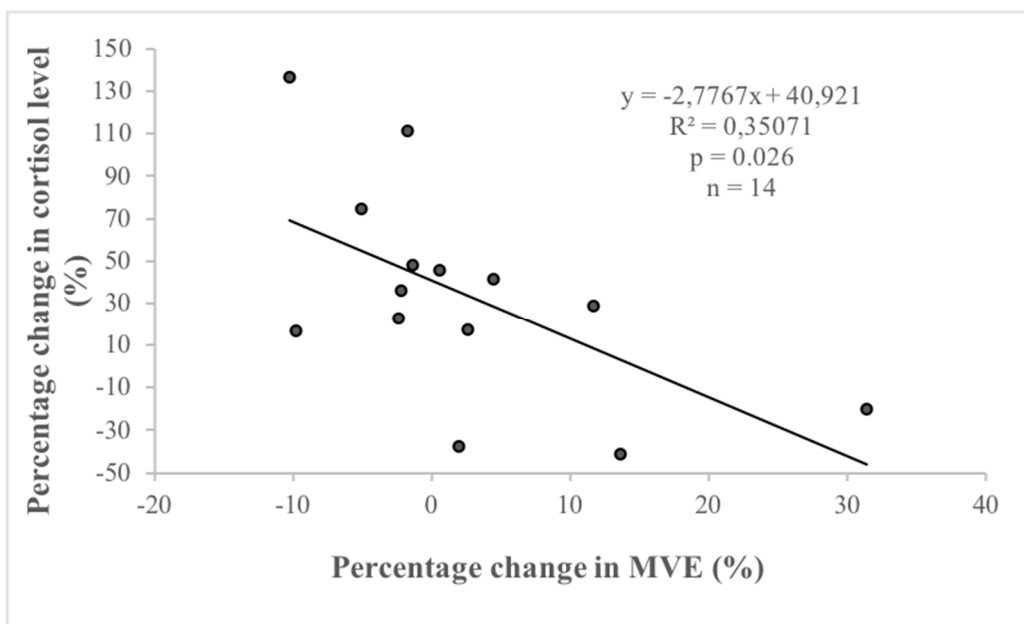

Figure S2

Supplement: Supplementary file 1 [file ijerph-18-09977-s001.zip › ijerph-1284816-supplementary.pdf]
